# “They just don’t have the “doctor” in front of their name:” Dimensions of trust of physician assistants

**DOI:** 10.1016/j.qrmh.2025.100018

**Published:** 2025-07-28

**Authors:** Staci Defibaugh, Leah Onosato

**Affiliations:** Old Dominion University, Norfolk, VA, USA

**Keywords:** Communication, Humanistic approach, Patient-centered care, Provider type preferences, Thematic analysis

## Abstract

Physician assistants play a major role in healthcare delivery in the United States, yet what we know about how patients perceive the care they receive from PAs is limited. Prior research on patients’ impressions of PAs has focused primarily on survey data, limiting the scope of what we can learn about patient impressions to predetermined, quantifiable categories, and has focused on post-visit impressions of a single encounter. In an attempt to better understand patient impressions, we conducted open-ended, semi-structured interviews with 30 participants prior to their medical visit, focusing on general impressions of PAs. Through an analytic process of reflexive thematic analysis, we identified three themes from the interview data: patients are confident in PAs, patients feel valued by PAs, and patients appreciate the openness that PAs create. Through exploration of these themes, we uncovered the ways in which interviewees described PAs as engendering trust and enacting multiple aspects of patient-centered care.

## Introduction

### Physician assistants/associates

Physician assistants (PAs), also known as physician associates, are medical providers who provide medical care to patients in the United States as well as many other countries (American Academy of Phyisican Associates [n.d.]). PAs have been part of the medical landscape in the US since 1968, when the first class of graduates from Duke University’s PA program graduated and entered the workforce (American Academy of Phyisican Associates [n.d.]). The creation of the profession grew out of a primary care physician shortage and has continued to grow, in part, because of that shortage. PAs, along with nurse practitioners (NPs), are commonly referred to as advanced practice providers (APPs) and are able to provide a wide range of medical care including diagnosing and treating medical conditions (National Cancer Institute [n.d.]). Across the US, PAs also have the ability to prescribe medications to patients, but in most states, they are required to work under the supervision of a physician. According to the US Bureau of Labor Statistics, in 2023, 145,740 PAs were practicing in the US, primarily in physician offices (Bureau of Labor Statistics, 2023), with expectations for the number of PAs to continue to grow by 26.5 % between 2022 and 2032, making the likelihood of a patient seeing a PA (or an NP) quite high. Therefore, understanding patient impressions of PAs provides insight into the work they are currently doing specifically within this changing medical landscape.

While the roles and training of NPs and PAs are similar, they differ drastically from medical doctors (MDs) and doctors of osteopathy (DOs). NPs can earn either a master’s or doctoral degree in nursing, requiring two to three years for the former and up to five years for the latter (Wintemute, 2025). PAs earn a master’s degree through a physician assistant program, often associated with a medical school, which typically takes two to three years to complete (Wintemute, 2025). Both programs require clinical rotations across a variety of specialties, and both fields require that professionals pass a qualifying exam. PAs, along with doctors and NPs, also have to complete annual continuing medical education hours in order to maintain their certifications (American Academy of Phyisican Associates [n.d.]). MD and DO educational programs are much more extensive, requiring four years of combined classroom and clinical training followed, minimally, by a three-year residency requirement (Cleveland Clinic, 2023). This difference in training and education level is reflected in the titles of doctors and lack of a similar professional title for NPs and PAs as well as the common, but dispreferred, term of “mid-level provider” which positions them as somewhere between a doctor and registered nurses.

### Patient perceptions of PAs

The first studies to examine perceptions of PAs came shortly after the profession came into being in the 1970s (Strunk, 1973, Jolly, 1980, Smith, 1981). These studies were primarily focused on patients’ acceptance of PAs and their willingness to be seen by a PA despite having little to no knowledge of the profession and likely no prior experience with them. Researchers reported that survey respondents were open to PAs performing “routine” care, but not necessarily more complex medical tasks (Strunk, 1973, Jolly, 1980, Smith, 1981). Strunk (1973) noted that as participants moved through the survey, they were more likely to report greater confidence in PAs, particularly when qualifiers such as “if they know what they are doing” were added. While mentioned, but not expanded upon by Strunk, the implication is that as knowledge of the profession increased throughout the completion of the survey, patients expressed a greater willingness to see PAs. From this, we might speculate that through exposure and direct experience with PAs, the likelihood of more favorable assessments of PAs will increase.

A number of research studies outside the US where PAs have been more recently incorporated into the healthcare system have also found generally positive impressions of PAs (Joyce et al., 2018, Halter et al., 2017, Meijer and Kuilman, 2017). Halter and colleagues (2017), for example, found varying positions across four identified themes which highlighted the differences in patients’ perspectives and experiences; however, they note that patients reported largely positive reviews of PAs practicing in England. Similarly, in a study conducted in the Netherlands, Mejier and Kuilman (2017) found equally high satisfaction rates by patients who saw a PA compared to patients who saw a physician.

More recent research exploring patient satisfaction in the United States has focused on comparisons of different provider types: PAs, NPs, and physicians. In a meta-analysis of 25 journal articles, Hooker, Moloney-Johns, and McFarland (2019) found no significant differences across the three groups, with survey respondents all reporting fairly high satisfaction ratings. Hooker et al. (2005) surveyed over 146,000 Medicare recipients about their primary care provider (PCP) on four measures of satisfaction: time spent, showing respect for patient’s input, explanations by provider, and listening. They found no differences across the three provider types. They also found no statistical differences based on sociodemographic factors of the survey respondents. In a similar study drawing on patient satisfaction surveys, Roblin and colleagues (2004) noted equal to or higher satisfaction rates for PA/NPs (they combined these two provider types); specifically on the metric of “interaction,” respondents reported higher satisfaction ratings for PA/NPs, suggesting that patients recognize the interactional work that PAs and NPs engage in during medical visits. It is important to note that the data from this study come from a managed care organization that triages patients based on their medical condition, meaning that PAs and NPs primarily saw patients with acute, rather than chronic, conditions; thus, the data do not necessarily address how people feel about seeing a PA for something more than “routine” care or whether they simply see PAs as one part of their healthcare team.

Taylor et al. (2019) took a more qualitative approach by conducting interviews with patients and/or their representatives who had just had an interaction with a PA in the hospital. They found overall positive evaluations of PAs across different themes including feelings of emotional support, confidence, sharing of information, and discussing treatment and illness management. One potential challenge in this study was that many of the interviewees did not realize they had seen a PA rather than a physician, which, the authors note, could erode trust if patients feel that it is not made clear. Nevertheless, when asked about their experiences with this single encounter, patients reported positive assessments of their PA visit.

More recently, as PAs and NPs have begun serving as primary care providers (PCPs), Leach et al. (2018) sought to address the question not of acceptance of APPs, but of potential preference for them. They asked survey respondents to imagine that they had to select a new provider and to identify whether they would prefer a physician or an APP (framed as PA/NP). While over half indicated that they would prefer a physician, 21 % reported a preference for a PA/NP, and the remaining 23 % said they had no preference. These data indicate a shift from earlier reporting in which patients would “accept” seeing a PA in only limited situations; in this case, it was clear that their prior experience with PAs and NPs led about one-fifth to prefer them over physicians. This study also included a qualitative element in which they asked respondents to indicate their reasons for their choice; those who preferred a PA or NP noted “bedside manner” and convenience as the two most cited factors.

The current study adds to this literature on patient satisfaction and impressions of PAs by taking a purely qualitative approach through semi-structured interviews. In doing so, participants were given the space to share any impressions they have rather than ones that fit within preconceived categories imposed by researchers. Additionally, we seek to add to prior research by asking about general impressions, allowing interviewees to draw on all prior interactions and report on their experiences with PAs more generally rather than a single interaction with a single provider.

### Patient-centered care, patient-centered communication, and person-centered care

Attributes of PAs (and NPs) that patients cited in previous studies, including “bedside manner,” “interaction,” and offering emotional support are associated with patient-centered communication. Patient-centered communication is sometimes conflated with, and sometimes viewed as a component of, the larger concept of patient-centered care, both abbreviated as “PCC.” Within the literature of patient-centered care/communication, a variety of related, but slightly differing, definitions exist. Key concepts across these definitions are that PCC typically involves a focus on 1) showing respect for patients’ experiences, 2) engaging in dialogue with patients through active listening and clear, approachable explanations, and 3) including patients in decision-making (Stewart et al., 2000, Epstein and Street, 2011, Davis et al., 2005, Hong and Oh, 2020). While some definitions include aspects such as access to medical records for patients or coordination of care (Davis et al., 2005), others frame PCC as a purely a communicative aspect of healthcare.

The fact that these terms have become conflated and, at time, synonymous, illustrates the importance of communication in healthcare delivery. Further complicating the terminology is the more recent framework of person-centered care, which, while overlapping with patient-centered care in many ways, may differ slightly in its focus or goals (Håkansson Eklund et al., 2019). Because person-centered and patient-centered care both align with the concepts that participants discussed, including displays of empathy and respect for the patient, treating the patient holistically, and engaging in shared decision making, we consider them to fall within a single framework of PCC.

Within definitions of PCC, as noted, researchers often point to the multi-faceted communicative aspects that are involved. Wanzer et al. (2004), for example, refer to PCC as “the *array* [emphasis added] of communicative behaviors that can enhance the quality of the relationship between the health care provider and patients, or the patient’s family” (p. 364). Hong and Oh (2020), drawing primarily on Epstein et al. (2005), operationalize PCC as

patient-provider communication that enhances patients’ comprehension (by explaining medical terms using layperson’s terms and by giving patients sufficient opportunity to ask questions) and perceived empathy (by listening attentively to patients and responding appropriately), and the encouragement of patient involvement and empowerment (by involving patients in decision making). (p. 504)

This definition divides PCC into three aspects: attending the patients’ informational needs, attending to patients’ emotional needs, and engaging in shared decision making, similar to findings elsewhere associating PCC with positive health outcomes (Yu et al., 2023) including patient self-efficacy (Finney Rutten et al., 2016).

### Trust as a reflection of PCC

Within the research on PCC, many scholars point to the necessity of trust as key to both to a productive provider-patient relationship (Croker et al., 2013, McKenna et al., 2020, Sharp et al., 2015) as well as to positive health outcomes for the patient (Birkhäuer et al. (2017); Lee & Lin, 2011). As Croker and colleagues note, “trust stems from the patient’s beliefs that the doctor is their ally and is competent in both clinical and interpersonal skills” (2013, p. 2). They further note that in order to create a trusting relationship, providers must engage in patient-centered approaches including shared decision making, effective and meaningful communication, and longer visit times. Others (Sharp et al., 2015, McKenna et al., 2020) have correlated trust with active and empathic listening and feeling known by their providers. Therefore, viewing participants’ impressions of PAs through the lenses of patient-centered care and trust is a fruitful endeavor. For these reasons, we seek to answer the following questions: 1) What are participants’ impressions of physician assistants? 2) How do these impressions align or disalign with the tenets of PCC and the concept of trust?

## Methods

### Data collection

Data for this paper are based on semi-structured interviews with patients in various medical offices in the Southeastern United States. These interviews are part of a larger project involving audio recordings of interactions between physician assistants and patients in various specialties, interviews with patients (the focus of this manuscript), and interviews with providers. For the purposes of this project, we chose to focus solely on the voices of patients rather than attempting to correlate the patient interviews with the other data sets.

The research project was approved by the Institutional Review Board (IRB) at Old Dominion University. The first author of this paper was solely responsible for all data collection including consenting and conducting the patient interviews.

### Participants and recruitment

PAs were first recruited to participate in the overall study; once a PA had consented to participate, arrangements were made between the first author and the medical office to arrange data collection days. Any patients who had scheduled appointments on those days were invited to participate in the study. In this way, sociodemographic factors such as age, gender, employment, or insurance status were not considered as factors for participation, nor were there efforts to create a representative sample of participants. One reason for this is that quantitative studies examining patient impressions or acceptance of PAs found no significant differences across demographics (Roblin et al., 2004, Berg et al., 2012, Meijer and Kuilman, 2017), and, in fact, Klinkenberg et al. (2011) argue that “demographic characteristics have been overrepresented in research on patient perceptions of care” (2011, p. 350). Additionally, because of the small number of participants in this study, generalizations based on demographic factors are not possible.

Participants were recruited from four different offices where participating PAs were working. Two were primary care offices where the PAs specialized in internal medicine. The other two were specialist offices: one in neurology and one in neurosurgery. Sites and specialties were selected based on the willingness of PAs to participate in the study. Because of this, the data is not representative of the scope of PA practice as a whole; however, there is representation in terms of primary care (i.e. internal medicine) and specialist care (neurology and neurosurgery).

A total of 30 participants were interviewed prior to their scheduled visit with a PA. In each case, they were asked if they had seen a PA before; all of them confirmed that they had. There were eight participants at each of the two internal medicine clinics, seven from the neurology clinic, and seven from the neurosurgery clinic. Each participant was given a unique identifier number consisting of the provider number and participant number, for example, 01–01 is PA 1-Participant 1. We will use these numbers to reference participants’ responses in the following sections.

### Interviews

Interview questions focused on two main topics: 1) prior experiences with and perceptions of physician assistants (and, to a lesser extent, nurse practitioners and physicians) and 2) positive and negative attributes of providers more generally. Participants were interviewed prior to their scheduled medical visit in order to gather general impressions rather than focusing on the current visit and current provider. In some cases, participants were seeing a PA they had prior experience with; for others it was their first visit with this provider. This led to different kinds of responses, with some moving from general impressions to talking about their specific PA quite quickly while others spoke more in generalities. Despite this, we believe that the responses to the questions represent interviewees' perceptions of PAs and their values regarding medical care and medical providers’ communicative practices.

Interviews ranged between one minute, 47 seconds to 17 minutes, 37 seconds, with the average length being 7 minutes, 22 seconds. Over half—18 of the 30—were in the five-to-nine-minute range. While the same basic questions were asked and the same amount of time was allotted for each interview, some participants offered very little in response to the questions, while others shared stories of past experiences and provided detailed answers. Interview questions varied depending on the responses, but generally followed the same format and included the same basic questions: 1) What are your general impressions of PAs? 2) If you are given a choice between a PA, an NP, or a physician, do you have a preference? 3) When it comes to a provider, what is really important to you? What are the things you really care about? 4) Have you ever had an experience with a provider that made you not want to see them again? 5) Is there anything else you want to share?

### Data analysis

Once all interviews were collected, the second author transcribed each of them. This ensured that both researchers began the coding and analysis process with some level of familiarity with the data. Data analysis followed a reflexive thematic approach, as outlined by Braun et al. (2022). A reflexive thematic analysis approach takes “a ‘big Q’ qualitative approach” (Braun & Clarke, 2021, p. 39) in which the research is both conceptualized and carried out within a framework that acknowledges researcher subjectivities, and rather than seeking to minimize these, embraces the background and positionalities that we bring to the study. In considering various qualitative approaches, Braun and Clarke (2021) suggest that reflexive thematic analysis is well-suited for studies that seek to identify themes that can encapsulate a multitude of voices—as opposed to delving deeply in to singular experiences—and that the focus is on how personal experiences can be understood and positioned within larger social or cultural contexts. As mentioned, interviewees who participated in this study had varying knowledge of and prior experiences with PAs, and it was important for all of those voices to be included.

Braun et al. (2022) outline the five steps of reflexive thematic analysis: 1) familiarization, 2) coding, 3) initial theme generation, 4) reviewing and developing themes, 5) refining, defining, and naming themes, and 6) producing the report. As mentioned, as first and second authors, we both initially familiarized ourselves with the data in different ways; however, we both also spent time reading through the transcripts before any coding or discussion began. Following the principles of reflexive thematic analysis, we focused less on structuring and constraining the coding process via a coding manual and instead followed a process of the applying codes inductively, while continuously reflecting on the process and our respective subjectivities. We initially coded the data separately while engaging in reflective discussions about the data and the codes throughout. Following initial coding, we then compared our coding, noting similarities and differences. From there, we began to identify themes that roughly aligned around the concepts of roles and expectations for the medical visit.

## Results

Our themes were derived from examination of the interview data and reflect overarching claims that participants made. The majority of participants expressed positive feelings toward PAs along the dimensions discussed below. Of the 30 interviewees, only four indicated that they would prefer a doctor. Three of these were patients at the neurosurgery clinic and who, as one person described herself, had more “complicated” (04−06) medical conditions that required a doctor’s expertise. The final person who indicated a preference for a doctor had a doctor of osteopathy (DO) as his primary care provider, but because of availability, was seeing the PA instead. The remaining 26 participants indicated either a lack of preference, viewing PAs as equally competent as doctors, or explicitly noted a preference for a PA or an NP, suggesting an implicit level of trust.

The three main themes we identified are: 1) patients are confident in PAs; 2) patients feel valued by PAs; and 3), and patients appreciate the openness that PAs create. Within each of these, we identified subthemes, expressed as first-person statements that reflect the participants’ articulations of their impressions of PAs. Trust in PAs can be seen in the articulation of each of the themes and subthemes (Table 1).

**Table 1:** Main themes and sub-themes.

Main theme	Sub-themes
Patients are confident in PAs	I am confident in PA’s abilities I am confident in PAs’ level of knowledge or expertise I feel confident because PAs seek answers A PA’s level of education does not diminish my confidence in them
Patients feel valued by PAs	I don't feel rushed I feel invested in
Patients appreciate the openness that PAs create	I am willing to share with PAs because they are approachable A PA’s level of education contributes to my feelings of comfort

### Theme 1: Patients are confident in PAs’ abilities

One of the clearest ways that participants conveyed a sense of trust in PAs was through expressed confidence in PAs’ abilities. This was described both in general terms as well as specifically related to professional knowledge or technical abilities. Two participants specifically used the term “trust,” while others used terms like “confidence” and “comfortable.” For example, one participant described a general trust in seeing PAs: “I’ve been to PAs most of the time, and I’m very comfortable with that” (02−03). Others spoke specifically about specific PAs, explaining, “I’m comfortable with the decisions she makes” (02−08) and “I have a lot of confidence in her” (02−07). Other participants described the care that PAs provide as being “thorough,” for example, “I feel right now that they are more thorough” (02−05). Thoroughness suggests a comprehensive or detailed approach to care. While these participants did not specifically use terms directly associated with confidence, describing one’s provider as “thorough” suggests a high level of competency, which can equate with confidence and trust.

Participants identified ways in which they view PAs as knowledgeable. For example, one participant explained, “My PA is pretty knowledgeable.... It seems like she just has a lot of knowledge about details of a medicine or a treatment” (02−06). Highlighting PAs’ knowledge reveals the value that this patient places on the PAs’ technical competency.

Others drew more explicit connections between a PA’s knowledge and their willingness or ability to share that knowledge with the patient, as well as the patient’s level of trust in their PA. For example, in comparing a PA to a physician, one participant focused not on the amount of knowledge that the PA displayed, but the amount that was shared with her:

The PA I see with my cardiac care, I like her a lot. She explained a lot more than the doctor did. I got his version of what was going on, and I got her version with more supportive information (04−05).

While this participant does not specifically link the amount or the quality of information to the idea of trust, there is an explicit statement of valuing or liking the PA. Similarly, we found that participants often described feelings of confidence in their providers because of their “more detailed” responses that provide options rather than simple diagnoses (02−02). Another patient encapsulated this connection between knowledge and confidence quite succinctly: “She doesn’t have to be like, ‘I’ll get back to you.’ She knows the answer, so I feel very comfortable with the decisions she makes” (03−02).

While some participants (such as 03–02, above) described confidence in their PA in terms of readily having answers, others described the feeling of confidence stemming from PAs’ seeking answers or solutions beyond their own scope of knowledge or in their willingness to explore alternative paths. One participant described a situation in which she had not gotten answers to an ongoing problem as an inner dialogue with herself about why she felt strongly about bringing this issue to her PA: “So I said, ‘I’ll work with somebody who I could get along with, work with, somebody who I trust to look into it’.... So I’m glad I’m with her" (03−03). This patient specifically describes the trust she feels in working with her PA as she juxtaposes her experience with this PA to her previous providers. While she seems to understand that her PA may not have the answers, she trusts her to ”look into it.”

Other participants included positive descriptions of PAs as exploring different solutions to help their patients. One person explained, “And if it doesn’t work or whatever, I don’t think she has a problem saying, ‘Well, we’ll try something different,’ and that part I think is great” (03−08).

Similarly, another participant described her PA as making the effort to find information that is relevant to her as a patient:

One of the most important things that I like about [my PA] is that she stays on top of things that are relatable to me, like menopause, and things that I’m going through. She does the reading. She looks outside of just her practice to find solutions. (02−05)

In this example, the participant explains how she values her PA being knowledgeable about issues that are relevant to her, demonstrating a personalized level of care, and seeking additional information when needed, suggesting that the PA does not rely only on her current knowledge of a health topic, but is continuously adding to that knowledge in order to better address patient needs.

Many were unequivocal that their confidence in PAs was based on the perceived skills of PAs and that they were unconcerned with the fact that PAs are not “doctors.” As one patient described, “They are very capable. The ones I’ve seen, it is just like a doctor, you know, and they just don’t have the ‘doctor’ in front of their name, but…they’ve all been very good” (03−01). Others expressed similar notions, suggesting that PAs are “just as qualified as a doctor” (04−02) or that they “know as much as the doctors” (04−05).

One participant provided a more in-depth explanation of why they view APPs as potentially being *more* competent that doctors:

I think a lot of people think because they're a PA or nurse practitioner, they’re not as knowledgeable as an MD. Sometimes, I kind of feel like they might be a little bit more knowledgeable because they have to be up to date. They see a lot more patients than the MDs do. They have to know the latest and greatest…the research .... So they they're usually on top of things a little bit more. (04−07)

All of these articulations of PAs’ level of knowledge suggest that participants view PAs as being competent and capable medical providers who have a great deal of knowledge, that they share this knowledge with patients by providing detailed explanations, that they are willing to seek answers when they do not have them readily at hand, and that their knowledge is not limited by lack of the designation of “doctor.” Overall, the majority of those interviewed either indicated they had no preference for provider type or that they preferred an APP, further indicating that most participants have confidence in APPs generally, and often, PAs, specifically.

### Theme 2: Patients feel valued by PAs

Participants frequently discussed a sense of feeling valued in their interactions with PAs—specifically, that they felt invested in and listened to. Codes for this theme included references to taking or spending time, thoroughness both in listening and explaining, and expressions of value.

Taking the time to listen to a patient or allowing a patient to share their concerns is associated with higher levels of trust (Sharp et al., 2015), and “listening” is a criterion that many participants discussed as something they felt was critical in their evaluation of providers more generally. According to McKenna and colleagues (2020), listening is a key factor in the development of trust. For participants in this study, feeling listened to was often equated with acknowledging the patient’s experience of illness and their personhood. In their work on person-centered care in nursing, Sharp et al. (2015) argue that acknowledgement of personhood and personal value are key to providing compassionate care to patients.

Participants described feeling invested in as a person more generally and as a patient more specifically, with PAs. For example, “I don’t feel that I’m just a number. I feel like that she takes the time to understand and listen to what I have to say” (02−04). The concept of feeling like “just a number” was something that many participants noted and likely reflects what otherwise can be a general feeling of depersonalization and dehumanization that many feel in the US healthcare system. For this participant, personalized care was associated with the provider listening and working toward ensuring that the patient is understood. It is also associated with taking the time that is needed to listen carefully, a topic that will be addressed more thoroughly in the discussion of the next subtheme.

Another participant described their experience as, “I feel like I’m listened to. I feel more engaged as the patient. I feel like they are invested in my health as a patient” (02−05). In this example, the participant describes how the PA can create a feeling of concern for the patient’s wellbeing and health. She equates listening with engagement and investment, all of which show a particular value of the patient by the PA. Others described feeling a sense of investment in themselves even beyond their role as a patient, for example:

I lost my husband a little over a year ago, and [PA] knew what I was going through. And she either she would email me through the portal or she would call me just to see how I was. You know, that's pretty outstanding. It makes you feel valued.... Yeah, and I guess feel seen in some ways right. (03−06)

Here, the participant describes a PA going beyond just showing concern within the visit or a concern with respect to an illness, describing a PA who is invested and concerned for the patient more holistically. The participant also explains how this made her feel both “valued” and “seen,” both of which suggest being acknowledged as a whole person with life experiences beyond their immediate health circumstances.

Another way participants described feeling valued was in not feeling rushed through the visit or feeling like the PA was able to spend as much time as was necessary. For example, one participant said, PAs “take more time with you. They explain it until you are fully satisfied. They are accommodating. They spend more time” (03−08). The idea of taking more time in this response is aligned with providing detailed explanations. Additionally, this participant framed the idea of spending more time in terms of taking as much time as the patient needs in order to feel “fully satisfied.”

Another patient described their PA as someone who “will sit and talk with me forever” (02−07). “Forever” is an example of an extreme case formulation (Pomerantz, 1986), acting as a sort of superlative, indicating how strongly this participant feels with regards to not feeling rushed through the visit. Others compared their experiences with PAs and physicians: “I feel like they have more time to spend than the physicians” (02−05); and “Generally, I feel they take more time with me than a doctor would” (02−01). This explicit comparison to physicians arose in the interviews many times (further discussion of this in the subsequent sections), even though no questions asked participants to compare PAs to doctors. Instead, participants were likely drawing on prior knowledge of visits with different provider types as well as positioning doctors as the default, or prototype, for medical providers.

Researchers have noted that it is not the case that APPs spend more time with patients than doctors (Morgan et al., 2014). Similarly, providers within the medical group where the interviews were collected also indicated that PAs, NPs, and physicians are allotted the same amount of time per visit. What this suggests is that the way that PAs interact with patients and the way they show investment and a willingness to take the time needed with the patient gives *the impression* of a greater time commitment. This is supported by claims from McKenna and colleagues (2020) who believe that active listening—being present and focused on patients—can lead to more efficient visits. It is quite possible, then, that PAs, through their communicative choices, create a sense of a *quantitative* difference in time, when in actuality it is a *qualitative* difference in how this time is spent.

### Theme 3: PAs Creation of a positive and inviting space

Participants often discussed how PAs were “easy to talk to” or described PAs as “approachable,” “understanding,” and/or “non-judgmental.” While all of these descriptions have slightly different connotations and may relate to somewhat different interactional practices, collectively, they point to participants’ experiences with PAs ability to create a comfortable space in which patients feel able to share their concerns or experiences. McKenna and colleagues (2020) link this to the act of listening which leads to trust:

Indeed, if listening builds trust and rapport, then the more time spent on listening may not only improve the breadth and quality of provider-patient relationships, it may also lead patients to share vital information that otherwise would not be disclosed. (2020: 375)

One participant described PAs as having “bedside manners” and explained that they are “more personable and easy to talk to” (01−01). Others used similar descriptions including feeling “more comfortable” with their PA because she is “easy to talk to” (02−07). One of the most compelling responses was from a participant who explained how her PA creates a context in which she is able to share:

It makes me want to share. You know, it makes you want to open up. It makes you want to share.... I've told [my PA] things that I don't think I've ever mentioned to any other provider ever. (02−05)

This participant specifically connected their desire to share with the theme of feeling invested. This suggests that when a PA shows investment in the patient, it alters what the patient might view as an allowable contribution. This likely relates to the idea of not feeling judged or feeling as if what the patient might share would be dismissed.

Further, the use of the extreme case formulation of “ever” indicates a drastically differing experience compared to other providers. In considering why this characteristic might be valued so much by participants, we reflect on a comment that another participant made: “Being a patient is always uncomfortable. I would just rather not be here” (02−03). Acknowledging that the medical encounter is space in which many might feel similarly, getting patients to feel comfortable enough to share “anything” (02−08) or something they’ve never shared with anyone else can certainly lead to better health outcomes, as providers would be able to gain additional insights into patients’ experiences that might lead to diagnostic opportunities or greater willingness or ability to follow a prescribed treatment plan.

Participants not only claimed that PAs are easy to open up to, but also discussed reasons for this. For example, one participant viewed PAs as having a greater ability to relate to the patient experience:

They've been where I'm at as a patient, and they seem to remember that experience. You know, nothing against MDs, but they've [PAs] worked their way to get where they're at, and I just seem to feel like they remember what it's like to be sitting here. (03−06)

While the participant did not provide an explanation of why she feels as though PAs “remember” the experience of being a patient, she seems to believe that this is related to their paths through PA school compared to medical school. The inference that PAs can relate to patients, no matter what the reasoning is, seems to be based on experiences with PAs and medical doctors.

Another participant related this difference to the use of titles compared to first names: “I just like them because they're easy to talk to. They don't mind you calling them by their first name, and that feels easier and more comfortable” (01−01). In the United States, within medical settings, only doctors are addressed by title plus last name. All other medical providers, including PAs, are typically addressed by first name only. As Hook (1984) explains, the use of title plus last name invokes a power dimension that is associated with hierarchy and social distance. The use of first names, alternatively, is associated with solidarity. This participant thus articulates how difference in naming between doctors and PAs creates a lowered sense of social distance and more equal footing, contributing to the feeling of comfort and ease.

## Discussion

The results of our reflexive thematic analysis reveal insights into how participants think about the care that PAs provide, indicating a level of trust present in a number of ways. The first theme most aligns with our understanding of trust, as confidence and trust are often used collectively or interchangeably. However, we have followed Croker and colleagues (2013), as well as others, who view trust as being created through a variety of means, including, but not limited to, confidence. Within this theme, we noted how participants discussed the confidence and comfort they have in seeing PAs and their impressions of PAs as being knowledgeable. In this way, the confidence that participants expressed seems to be coming from the technical skills that PAs possess more so than the interpersonal skills. When thinking about the components of patient-centered care that Hong and Oh (2020) describe, we might align this with informational needs of patients. Similarly, participants often described PAs as being “just as” or “even more” knowledgeable or competent as doctors, creating an explicit comparison. This suggests that, for many patients, a PA’s level of medical degree is not the determining factor in their confidence. Instead, it comes from knowledge displayed and a willingness to seek additional information when necessary.

Second, participants described PAs showing value to them as patients and as individuals. This was described as PAs engaging in listening practices that made participants feel that their concerns were being heard and acknowledged and PAs taking as much time as needed to both listen and explain. Sharp, McAllister, and Broadbent align both of these aspects of care with increased trust, noting that being known, or as one participant described it, feeling “seen,” is a “source of trust and confidence in care” (2015: 6) and that not being rushed can lead to validation or feelings of self-worth. Both of these are essential aspects of patient-centered and person-centered care, as they acknowledge personhood and show value for patients.

Finally, the third theme most closely reflects the vulnerability that is inherent in medical encounters (Croker et al., 2013) and the ways that PAs might create a space that both acknowledges and lowers that sense of vulnerability, allowing patients to open up and share potentially critical information. Being able to trust one’s provider means feeling more comfortable sharing personal or sensitive information. While this might reflect personal choices that specific PAs make, it also might, as one participant noted, be related to the lowered sense of hierarchy that comes from the use of first name rather than title plus last name. When hierarchies are diminished in dyadic exchanges, participants may feel a greater sense of closeness, which might, in turn, lead to a stronger and more meaningful provider-patient relationship.

It is important to note that any one of these three aspects of care could lead to greater sense of trust in PAs. However, it is likely that their enactment in combination and the ways that they coalesce in practice are most meaningful. For example, without having time to explain their concerns through engaged listening, patients might not feel they can open up and share deeper concerns. Similarly, if they are rushed through the visit, opportunities for explanations and knowledge sharing to take place is diminished. Trust in one’s medical provider is a cornerstone of effective, patient-centered care and one that participants in our study perceive in the ways that PAs practice medicine.

### Dissenting opinions

As mentioned above, four participants preferred a doctor over a PA or an NP. Two of the four provided no description of PAs or their impressions of them as qualified, competent, or trustworthy. Instead, they simply described doctors as having the expertise to handle complicated cases. One person who expressed a preference for a doctor explained that it depended on whether it was for a follow-up visit or a new condition, suggesting that for follow-up visits, seeing a PA was perfectly acceptable. This last response aligns with the early studies which often found that people would be willing to see a PA only if their medical condition was considered not to be very serious or to be just routine (Strunk, 1973, Jolly, 1980, Smith, 1981).

There was one outlier in the data who explicitly expressed a lack of trust in PAs, citing their title of “assistant” as leading to this lack of trust. As he explained, “A physician assistant to me is the doctor's helper, so why would I see the helper if I can see the boss” (03−04)? It is this impression that has likely led to the American Academy of Physician Associates to use the term “associate” rather than “assistant” to better reflect the roles they serve (American Academy of Phyisican Associates [n.d.]). While the responses of these four participants illustrate that not all patients have the same level of trust in PAs, the majority of those we interviewed expressed a level of trust in PAs that was either equal to or exceeded that of doctors.

## Limitations & future directions

Because we conducted interviews rather than surveys, the responses are not as neatly aligned to all aspects of PCC or components of trust, nor were we able to get equally through responses from each participant. As noted earlier, the length of interviews differed greatly depending on how much each individual wanted to share, and this is evident in the analysis where certain voices are more prominent than others. However, we feel that allowing space for participants to share as much or as little and for them to elaborate where they felt appropriate did provide nuanced data. We also found that participants who were seeing a PA as their primary care provider tended to provide longer, more elaborate responses. Also, because we only had three specialty offices represented, interviews from patients at a wider range of provider offices might lead to different results. Future research could include analysis of observations or recordings of medical visits from different provider types to triangulate the findings from this study and provide a deeper understanding of how PAs enact patient-centered care and engender trust in their patients.

## CRediT authorship contribution statement

**Staci Defibaugh:** Writing – review & editing, Writing – original draft, Methodology, Formal analysis, Data curation, Conceptualization. **Leah Onosato:** Writing – review & editing, Writing – original draft, Formal analysis.

## Declaration of interests

The authors declare that they have no known competing financial interests or personal relationships that could have appeared to influence the work reported in this paper.
